# Prevalence and incidence of chronic kidney disease stage 3–5 – results from KidDiCo

**DOI:** 10.1186/s12882-023-03056-x

**Published:** 2023-01-19

**Authors:** Jan Dominik Kampmann, James Goya Heaf, Christian Backer Mogensen, Hans Mickley, Donna Lykke Wolff, Frans Brandt

**Affiliations:** 1 Department of Internal Medicine, University Hospital of Southern Denmark, Sydvang 1, Sonderborg, 6400 Denmark; 2 Institute of Regional Health Research, University of Southern Denmark, Campusvej 55, Odense, 5230 Denmark; 3 Department of Medicine, Zealand University Hospital, Roskilde, Sygehusvej 10, Roskilde, 4000 Denmark; 4 Department of Emergency Medicine, University Hospital of Southern Denmark, Kresten Philipsens Vej 15, Aabenraa, 6200 Denmark; 5 Department of Cardiology, Odense University Hospital, J. B. Winsløws Vej 4, Odense, 5000 Denmark

**Keywords:** Chronic kidney Disease, Epidemiology, Prevalence, Socioeconomics

## Abstract

**Background:**

Chronic kidney disease (CKD) is a global challenge. CKD prevalence estimation is central to management strategies and prevention. It is necessary to predict end stage kidney disease (ESKD) and, subsequently, the burden for healthcare systems. In this study we characterize CKD stage 3–5 prevalence and incidence in a cohort covering the majority of the Region of Southern Denmark and investigate individuals’ demographic, socioeconomic, and comorbidity status.

**Methods:**

We used data from the Kidney Disease Cohort (KidDiCo) combining laboratory data from Southern Denmark with Danish national databases. Chronic kidney disease was defined according to the Kidney Disease: Improving Global Outcomes (KDIGO) guidelines.

**Results:**

The prevalence varied between 4.83 and 4.98% and incidence rate of CKD was 0.49%/year. The median age was 76.4 years. The proportion of individuals with CKD stage 3–5 in the entire population increased consistently with age. The percentage of women in the CKD 3–5 group was higher than in the background population. Diabetes mellitus, hypertension and cardiovascular disease were more prominent in patients with CKD. CKD stage 5 and ESKD were more frequent as incident CKD stages in the 18–49 year olds when compared to older individuals. CKD patients tended to have a lower socioeconomic status.

**Conclusion:**

Chronic kidney disease stage 3–5 is common, especially in the elderly. Patients with CKD stage 3–5 are predominantly female. The KidDiCo data suggests an association between lower socioeconomic status and prevalence of CKD.

**Supplementary Information:**

The online version contains supplementary material available at 10.1186/s12882-023-03056-x.

## Introduction

Chronic kidney disease (CKD) is a global healthcare burden [1]. As a major non-communicable disease, it is associated with adverse clinical and economical outcomes [2]. However, awareness of CKD is low [3]. Early detection is important to prevent and delay progression of CKD [2]. CKD is associated with increased risk of death, cardiovascular disease (CVD), and high healthcare costs [4]. The majority of CKD patients have diabetes (DM), hypertension (HT) and/or CVD, driven by a reciprocal relationship among these four major chronic diseases which complicates relevant treatment [5]. CKD is classified by Kidney Disease: Improving Global Outcomes (KDIGO) into 5 stages. Stages 1 and 2 require presence of kidney damage e.g. proteinuria [6]. Stages 3–5 are defined by glomerular filtration rate below 60 ml/min/1.73m^2^ over at least 3 months [6]. Stages 3 and 4 (GFR 59-15 ml/min/1.73m^2^) represent loss of 50% of normal kidney function and are seen as a cut-off for clinically significant CKD [7, 8]. CKD stage 3 is further divided into CKD stage 3a (59-45 ml/min/1.73m^2^) and 3b (44-30 ml/min/1.73m^2^). Stage 5 covers GFR under 15 ml/min/1.73m^2^.

Albuminuria measurement for kidney disease and cardiovascular risk stratification is recommended by current guidelines [6]. KDIGO guidelines divide the albumin/creatinine ratio into stages A1 to A3; albuminuria beneath 30 mg/g defined as normal (A1) to over 300 mg/g defined as severely increased (A3).

The crude prevalence of CKD in Europe spans from 3.3% in Norway to 17.3% in Northeast Germany [9]. Reliable data on local CKD prevalence is, therefore, challenging to estimate.

CKD prevalence estimation is central to CKD management strategies, also for prevention of end-stage kidney disease (ESKD) and subsequent cost to healthcare systems [10, 11].

The aim of this study is to establish the prevalence and incidence rate of CKD stage 3–5 in the Region of Southern Denmark, and explore the pattern of variation socioeconomically and demographically.

## Materials and methods

### Study population

Data was extracted from the Kidney Disease Cohort (KidDiCo) [12] of Southern Denmark. Patients 18 years+ whose creatinine was measured in one of 27 participating laboratories in the Region of Southern Denmark from 01.01.2006 to 31.12.2013 were included [12].

### Laboratory data

Laboratory data included inpatient, outpatient, and general practitioners’ practices data. All data are recorded according to unique personal 10 digit social security numbers, allowing record linkage with national databases.

### Assessment of kidney function

To estimate glomerular filtration rate (GFR), the Chronic Kidney Disease Epidemiology Collaboration (CKD-EPI) formula was used [6]. In case of same day multiple creatinine measurements, the highest creatinine value was used to estimate GFR. Creatinine was analysed with Jaffe and enzymatic assay. The Jaffe method is in excellent agreement with the enzymatic assay, leading to minimal differences only [12].

CKD was defined by one eGFR value < 60 ml/min/1.73m^2^ and a second eGFR value < 60 ml/min/1.73m^2^ measured at least 3 months later, however, no longer than 12 months apart. No eGFR > 60 ml/min/1.73m^2^ between both measurements was allowed, as recommended by KDIGO [6]. The earliest point of time where these criteria were fulfilled was defined as the individual CKD date (ICKDD) during 2007–2013. Henceforth, patients fulfilling CKD 3–5 criteria are referred to as CKD patients.

Different stages were defined according to KDIGO [6]. ESKD was defined as an ICD-10 code Z99.2 and/or Z94.0 according to The Danish National Patient Register (DNPR), regardless of eGFR. The respective CKD stages are based on the eGFR or ICD-10 codes at ICKDD or first available creatinine measurement (FACM).

### Albuminuria

Albuminuria was assessed 12 months from ICKDD or FACM. The amount of albuminuria is divided according to KDIGO guidelines into stage A1 = < 30 mg/g, A2 = 30–300 mg/g and A3 = > 300 mg/g [6]. The albumin/creatinine ratio measurement closest to ICKDD defined the CKD albuminuria stage.

### Control population

The control population was defined as residents in the defined geographic area at any stage between 2007 and 2013, with at least one creatinine measurement between 2007 and 2013, and who did not fulfil the CKD 3–5 criteria. Individuals living in the geographically defined area during the given time period are referred to as inhabitants.

### Prevalence and incidence rate

Calculations of prevalence and incidence rate were based on publicly available data from Statistics Denmark (https://www.statistikbanken.dk/statbank5a/default.asp?w=1280) on the number of inhabitants aged 18 years+ who lived in the defined region. Data was available quarterly from 2008.

To become prevalent, CKD criteria for CKD stage 3–5 had to be met at one stage within the time period from January 1st, 2006 to December31^st^, 2013 in accordance with ICKDD. Prevalence was defined as the number of accumulated cases alive per year in relation to all living individuals based on the entire population at the fourth quarter of the respective year.

Patients who for the first time fulfilled CKD stage 3–5 criteria between 2008 and 2013 were defined as incident cases according to the year of ICKDD. Patients fulfilling CKD criteria already in 2006–2007 were excluded to secure incident cases. Since creatinine is a common blood sample used in clinics and by general practitioners, we assume that most CKD 3–5 patients are identified during this 2 year period.

The different time periods used in our study are due to dependence on data from Statistics Denmark. Since a change of community coding data in residency occurred in 2007 in Denmark, Statistics Denmark were unable to provide data that could be linked with KidDiCo, despite the availability of blood samples since 2006. Data on the population of the entire region was first available from 2008 and onwards to calculate prevalence and incidence. For further clarification, an overview of the different availabilitiy dates of data can be seen in supplementary Fig. 1.

### Databases

The Danish Civil Registration System (DCRS) contains information on demographics, date of death, and residence of all persons living or having lived in Denmark [13].

The DNPR contains information on all diagnoses from somatic hospital wards and/or outpatient admissions [13]. Registrations of diagnoses are based on the International Classification of Diseases using ICD-10.

The Danish National Prescription Registry (DNPrR) holds information on all drugs sold in Danish community pharmacies according to the ATC (Anatomic Therapeutic Chemical)-code [13].

### Demographic data

Age was defined by ICD for patients with CKD 3–5 and by FACM for the control population. Sex was defined according to the last digit in the personal 10-digit number.

### Comorbidity data

Comorbidity was measured by the Charlson score (CS) based on information according to the ICD-10 [14]. We calculated the CS according to primary and secondary diagnosis from 10 years prior to FACM or 10 years prior to ICKDD respectively.

Diabetes mellitus (DM), hypertension (HT), and cardiovascular disease CVD- CS diagnoses were enriched with ATC codes. CVD was defined as ICD-10 codes for myocardial infarction, congestive heart failure, peripheral vascular disease or cerebrovascular disease. HT and DM diagnoses were enriched using redeemed drug prescriptions +/− 3 months from ICKDD. For HT, the following ATC codes were used: C03 “diuretics”, C07 “beta-blocking agents”, C08 “calcium-channel blockers”, C09 “agents on the renin-angiotensin system”. For DM, the ATC code A10 “drugs-used-in-diabetes” was used.

### Socioeconomic data

Educational levels were divided into short, middle, long, and missing data at time of FACM or ICD. Short educational level includes primary school, high school, and adult education. Middle education level includes bachelor degree or further education at bachelor level. Long education level includes higher education, research, and Phd. Missing information was stated as missing in the table. The afore-mentioned educational categories are based on recommendations from Statistics Denmark and are used in a similar fashion in a Danish previous cohort [15].

Occupational status was divided into “active”, “temporarily-not-active” including unemployed at least half of the respective year, sick leave etc.; “not-active” (NA) pensioners, individuals on welfare etc. All data are based on the respective year prior to ICKDD or FACM.

For the Tables 1, 2 and 3 a χ^2^-test was performed to test for differences between the groups.

**Table 1:** Baseline characteristics of patients with CKD stage 3–5 and the background population

		Total	Control population	CKD	***p***-value
		***N*** = 669,929	***N*** = 603,443	***N*** = 66,486	
Sex	male	309,757 (46.2%)	283,452 (47.0%)	26,305 (39.6%)	< 0.001
	female	360,172 (53.8%)	319,991 (53.0%)	40,181 (60.4%)	
Age		51.4 (37.1–65)	49 (35.8–61.6)	76.4 (69–83)	< 0.001
Age group (in years)	18–29	99,914 (14.9%)	99,777 (16.5%)	137 (0.2%)	< 0.001
	30–39	92,628 (13.8%)	92,306 (15.3%)	322 (0.5%)	
	40–49	120,927 (18.1%)	119,991 (19.9%)	936 (1.4%)	
	50–59	121,405 (18.1%)	117,841 (19.5%)	3564 (5.4%)	
	60–69	116,239 (17.4%)	103,046 (17.1%)	13,193 (19.8%)	
	70–79	70,987 (10.6%)	47,480 (7.9%)	23,507 (35.4%)	
	80–89	39,701 (5.9%)	19,129 (3.2%)	20,572 (30.9%)	
	90 and over	8128 (1.2%)	3873 (0.6%)	4255 (6.4%)	
Diabetes	no	634,636 (94.7%)	579,556 (96.0%)	55,080 (82.8%)	< 0.001
	yes	35,293 (5.3%)	23,887 (4.0%)	11,406 (17.2%)	
Hypertension	no	474,251 (70.8%)	461,510 (76.5%)	12,741 (19.2%)	< 0.001
	yes	195,678 (29.2%)	141,933 (23.5%)	53,745 (80.8%)	
Cardiovascular diseases	no	619,820 (92.5%)	572,643 (94.9%)	47,177 (71.0%)	< 0.001
	yes	50,109 (7.5%)	30,800 (5.1%)	19,309 (29.0%)	
Charlson	0	602,569 (89.9%)	557,058 (92.3%)	45,511 (68.5%)	< 0.001
	1	24,682 (3.7%)	18,759 (3.1%)	5923 (8.9%)	
	2	34,706 (5.2%)	23,348 (3.9%)	11,358 (17.1%)	
	3	3505 (0.5%)	1633 (0.3%)	1872 (2.8%)	
	4+	4467 (0.7%)	2645 (0.4%)	1822 (2.7%)	
Education level	short	487,400 (72.8%)	436,742 (72.4%)	50,658 (76.2%)	< 0.001
	middle	139,626 (20.8%)	132,909 (22.0%)	6717 (10.1%)	
	long	12,793 (1.9%)	12,396 (2.1%)	397 (0.6%)	
	missing	30,110 (4.5%)	21,396 (3.5%)	8714 (13.1%)	
Occupational status	active	327,358 (48.9%)	322,546 (53.5%)	4812 (7.2%)	< 0.001
	temporarily not active	16,962 (2.5%)	16,671 (2.8%)	291 (0.4%)	
	not active	305,147 (45.5%)	244,144 (40.5%)	61,003 (91.8%)	
	missing/others	20,462 (3.1%)	20,082 (3.3%)	380 (0.6%)

**Table 2:** Age stratification of CKD and background population

		CKD Age group 18–39 years	CKD Age group 40–69 years	CKD Age group 70+ years	Non-CKD Age group 18–39 years	Non-CKD Age group 40–69 years	Non-CKD Age group 70+ years	*p*-value
		***N*** = 459	***N*** = 17,693	***N*** = 48,334	***N*** = 192,083	***N*** = 340,878	***N*** = 70,482	
Sex	male	231 (50.3%)	7306 (41.3%)	18,768 (38.8%)	81,711 (42.5%)	169,909 (49.8%)	31,832 (45.2%)	< 0.001
	female	228 (49.7%)	10,387 (58.7%)	29,566 (61.2%)	110,372 (57.5%)	170,969 (50.2%)	38,650 (54.8%)	
Diabetes	no	393 (85.6%)	13,858 (78.3%)	40,829 (84.5%)	188,545 (98.2%)	324,963 (95.3%)	66,048 (93.7%)	< 0.001
	yes	66 (14.4%)	3835 (21.7%)	7505 (15.5%)	3538 (1.8%)	15,915 (4.7%)	4434 (6.3%)	
Hypertension	no	131 (28.5%)	4092 (23.1%)	8518 (17.6%)	184,282 (95.9%)	245,401 (72.0%)	31,827 (45.2%)	< 0.001
	yes	328 (71.5%)	13,601 (76.9%)	39,816 (82.4%)	7801 (4.1%)	95,477 (28.0%)	38,655 (54.8%)	
Cardiovascular diseases	no	413 (90.0%)	13,566 (76.7%)	33,198 (68.7%)	190,874 (99.4%)	322,931 (94.7%)	58,838 (83.5%)	< 0.001
	yes	46 (10.0%)	4127 (23.3%)	15,136 (31.3%)	1209 (0.6%)	17,947 (5.3%)	11,644 (16.5%)	
Charlson	0	336 (73.2%)	12,323 (69.6%)	32,852 (68.0%)	184,551 (96.1%)	314,578 (92.3%)	57,929 (82.2%)	< 0.001
	1	63 (13.7%)	1644 (9.3%)	4216 (8.7%)	4690 (2.4%)	10,038 (2.9%)	4031 (5.7%)	
	2	42 (9.2%)	2667 (15.1%)	8649 (17.9%)	2553 (1.3%)	13,779 (4.0%)	7016 (10.0%)	
	3	9 (2.0%)	466 (2.6%)	1397 (2.9%)	63 (0.0%)	824 (0.2%)	746 (1.1%)	
	4+	9 (2.0%)	593 (3.4%)	1220 (2.5%)	226 (0.1%)	1659 (0.5%)	760 (1.1%)	
Education level	short	356 (77.6%)	14,172 (80.1%)	36,130 (74.8%)	139,326 (72.5%)	244,048 (71.6%)	53,368 (75.7%)	< 0.001
	middle	84 (18.3%)	2840 (16.1%)	3793 (7.8%)	44,520 (23.2%)	80,962 (23.8%)	7427 (10.5%)	
	long	9 (2.0%)	177 (1.0%)	211 (0.4%)	4493 (2.3%)	7436 (2.2%)	467 (0.7%)	
	missing	10 (2.2%)	504 (2.8%)	8200 (17.0%)	3744 (1.9%)	8432 (2.5%)	9220 (13.1%)	
Occupational status	active	239 (52.1%)	4022 (22.7%)	551 (1.1%)	111,276 (57.9%)	209,774 (61.5%)	1496 (2.1%)	< 0.001
	temporarily not active	22 (4.8%)	269 (1.5%)	0 (0.0%)	7298 (3.8%)	9373 (2.7%)	0 (0.0%)	
	not active	191 (41.6%)	13,045 (73.7%)	47,767 (98.8%)	61,951 (32.3%)	113,252 (33.2%)	68,941 (97.8%)	
	missing/others	7 (1.5%)	357 (2.0%)	16 (0.0%)	11,558 (6.0%)	8479 (2.5%)	45 (0.1%)

**Table 3:** Baseline characteristics according to CKD stage in Patients with CKD

		Total	CKD 3a	CKD 3b	CKD 4	CKD 5	ESKD	*p*-value
		***N*** = 66,486	***N*** = 44,620	***N*** = 15,765	***N*** = 4359	***N*** = 1176	***N*** = 566	
Sex	male	26,305 (39.6%)	17,267 (38.7%)	6156 (39.0%)	1893 (43.4%)	657 (55.9%)	332 (58.7%)	< 0.001
	female	40,181 (60.4%)	27,353 (61.3%)	9609 (61.0%)	2466 (56.6%)	519 (44.1%)	234 (41.3%)	
Age mean		76.4 (69–83)	75 (68–81.4)	80 (73–85.6)	81 (72.4–87)	73.64999 (62.85–82.9)	62 (47–73)	< 0.001
Age group (in years)	18–29	137 (0.2%)	42 (0.1%)	26 (0.2%)	15 (0.3%)	17 (1.4%)	37 (6.5%)	< 0.001
	30–39	322 (0.5%)	119 (0.3%)	80 (0.5%)	46 (1.1%)	34 (2.9%)	43 (7.6%)	
	40–49	936 (1.4%)	553 (1.2%)	149 (0.9%)	82 (1.9%)	76 (6.5%)	76 (13.4%)	
	50–59	3564 (5.4%)	2600 (5.8%)	571 (3.6%)	172 (3.9%)	123 (10.5%)	98 (17.3%)	
	60–69	13,193 (19.8%)	10,267 (23.0%)	2027 (12.9%)	534 (12.3%)	237 (20.2%)	128 (22.6%)	
	70–79	23,507 (35.4%)	17,035 (38.2%)	4909 (31.1%)	1135 (26.0%)	298 (25.3%)	130 (23.0%)	
	80 and over	24,827 (37.3%)	14,004 (31.4%)	8003 (50.8%)	2375 (54.5%)	391 (33.2%)	54 (9.5%)	
Diabetes	no	55,080 (82.8%)	37,464 (84.0%)	12,848 (81.5%)	3429 (78.7%)	930 (79.1%)	409 (72.3%)	< 0.001
	yes	11,406 (17.2%)	7156 (16.0%)	2917 (18.5%)	930 (21.3%)	246 (20.9%)	157 (27.7%)	
Hypertension	no	12,741 (19.2%)	9872 (22.1%)	2108 (13.4%)	528 (12.1%)	201 (17.1%)	32 (5.7%)	< 0.001
	yes	53,745 (80.8%)	34,748 (77.9%)	13,657 (86.6%)	3831 (87.9%)	975 (82.9%)	534 (94.3%)	
Cardiovascular diseases	no	47,177 (71.0%)	33,424 (74.9%)	10,077 (63.9%)	2527 (58.0%)	763 (64.9%)	386 (68.2%)	< 0.001
	yes	19,309 (29.0%)	11,196 (25.1%)	5688 (36.1%)	1832 (42.0%)	413 (35.1%)	180 (31.8%)	
Albuminuria stage	A1	4942 (79.7%)	3667 (82.6%)	1028 (76.0%)	191 (62.4%)	22 (43.1%)	34 (66.7%)	< 0.001
	A2	1090 (17.6%)	687 (15.5%)	282 (20.9%)	93 (30.4%)	15 (29.4%)	13 (25.5%)	
	A3	168 (2.7%)	86 (1.9%)	42 (3.1%)	22 (7.2%)	14 (27.5%)	4 (7.8%)	
Albumin assesment rate	assessed	6200 (9.3%)	4440 (10.0%)	1352 (8.6%)	306 (7.0%)	51 (4.3%)	51 (9.0%)	< 0.001
	not assessed	60,286 (90.7%)	40,180 (90.0%)	14,413 (91.4%)	4053 (93.0%)	1125 (95.7%)	515 (91.0%)	

Stata version 16 was used for statistical analysis [16]. The manuscript was written in accordance with the STROBE statement [17].

## Results

Predominately, women suffered from CKD. The proportion of women was higher in the CKD-group (60.4%) than in the control population 53.0% (*p* < 0.001). The median age was higher in CKD patients than in the control population 76.4 vs. 49.0 years of age (*p* < 0.001).

In the control population 92.3% scored 0 in the CS compared to 68.5% in CKD patients - see Table 3.

CVD was 5.7 times more frequent in CKD patients (29.0%) compared to the control population (5.1%) (*p* < 0.001). DM was 4.3 times more frequent in CKD patients (17.2%) compared to the control population (4.0%) (*p* < 0.001). HT was common in both groups; 80.8% in the CKD group and 23.5% in the control population (*p* < 0.001).

Educational level in both arms showed a majority of individuals with short education; 76.2% in CKD patients and 72.4% in the control population (*p* < 0.001).

Social status data shows that 91.8% of CKD patients were “not-economically-active” compared to 40.5% in the control population (*p* < 0.001).

## Age and sex stratification for comorbidity and socioeconomic status

**Fig. 1:**
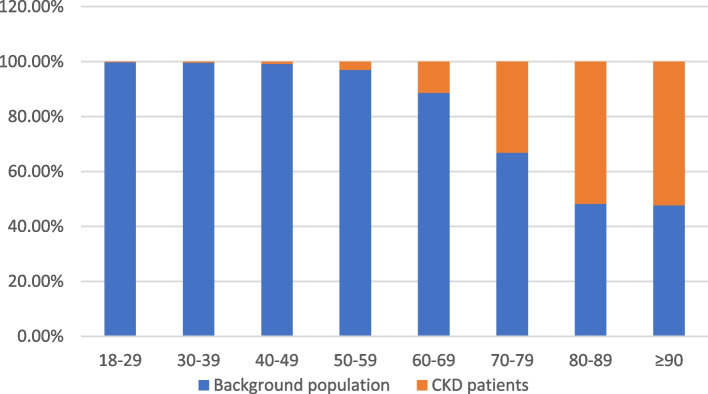
Age group stratification of incident CKD stage 3–5 patients and the Background population

Due to the evident age gap between CKD patients and the control population, we performed an age stratification into three age groups; 18–39 years, 40–69 years, and 70 years+ based on comparable CKD percentage per age group. In the 18–39 age group, less than 0.25% had CKD. In the 40–69 age group, mean percentage of CKD was 5%, and in the 70 years+ age group mean percentage of CKD was 46% (Fig. 1).

The increase of CSalong age groups was more prominent in the control population. This was also the case for HT and CVD, however not for DM. The proportion of NA in the 40–69 age group was 2.2 times higher in the CKD group, and in the 18–39 age group, the percentage was 1.3 times higher in the CKD group (Table 4).

Additionally, we performed sex stratification (see supplement Table 1). In CKD patients, the percentage of males decreases from 50.3% in18–39 year olds to 38.8% in the 70 years+ age group. Comorbidities were more prominent in males, especially regarding CVD and DM.

### Baseline characteristics according to CKD stage

Most patients with CKD become incident at CKD 3a (67.11%) or CKD 3b (23.71%). The median age in ESKD incident patients is lower at 62 years compared to other CKD stages where median age is between 74 (CKD stage 5) and 81 (CKD stage 4). The ratio of women is higher from CKD stage 3–4. This relation switches in CKD stage 5 and ESKD.

The percentage of patients with DM and HT increases concurrently with CKD stages at incidence. CVD percentage increased from 25.1% in CDK 3a and 42% in CKD 3b and decreased afterwards to 31.8% in ESKD.

Albumin/creatinine ratio data was available in 9.9% of CKD stage 3a patients and in 9.0% of ESKD patients. The percentage of available samples decreased concurrently from 9.9% in CKD stage 3a patients to 4.3% in CKD stage 5 patients rising sharply to 9.0% in ESKD patients.

The majority of patients presented with albuminuria stage A1. Moderate and advanced albuminuria were most prominent in incident CKD stage 5 patients with A2 29.4% and A3 25.5% (Table 1).

### Prevalence and incidence rate of CKD patients

The prevalence of CKD stages 3–5 patients between 2008 and 2013 varied between 4.83% (in 2008) and 4.98% (in 2009 and 2012) (Table 4). The prevalence did not show a significant change in the study period.

**Table 4:** Data on cases, inhabitants and prevalence

	2008	2009	2010	2011	2012	2013
Cases of CKD in KidDiCo	46,552	47,963	47,628	47,709	48,024	47,104
Inhabitants	962,348	962,638	963,070	963,781	964,105	965,207
Prevalence per year	4.83	4.98	4.94	4.95	4.98	4.88

All new cases with CKD stages 3–5 from 2007 to 2013 were summed up and divided with the sum of patients who lived in the geographical area from 2008 to 2013 in the fourth quarter of the respective year (0.49%)

## Discussion

Our study showed that CKD stage 3–5 is a common disease with a seemingly stable prevalence. Patients with CKD stage 3–5 are predominantly females, elderly, and with a higher comorbidity burden. Males have a higher CKD stage at incidence. Most patients with CKD stage 3–5 are not economically active and frequently have lower educational levels than the control population. Albuminuria testing is sparse throughout the cohort.

A predominance of women in CKD patients has been shown previously [2, 15, 18]. The higher proportion of men in CKD stage 5 and ESKD is similar to data from USA where incidence of ESKD was higher in men [19]. One explanation could be that CKD progresses faster in men [20].

Women are known to be more vigilant at following health recommendations and more likely to consult a doctor [21]. This might explain the higher proportion of females with incident CKD stage 3–4. CKD stage 5 and incident ESKD patients are more likely to be men possibly due to males consulting physicians later during their illness [21].

The median age of CKD stage 3–5 patients was significantly higher compared to the control population, which is similar to previous studies [2, 18]. The proportion of CKD stage 3–5 patients reaches a third of the population in 70–79 year olds and over half in the 80+ population. In the SCREAM cohort, the 85 years+ age group (50.9%) had CKD stage 3–5 which is comparable to our study results [18]. The decrease in the percentage of males in the CKD group with increasing age could be due to CKD patients dying before reaching the next age group. The higher proportion of comorbidities in men could be a result of unhealthier lifestyles and lower levels of compliance [22].

Our results showed significantly higher CS in CKD patients in accordance with other studies [18, 23]. Whether comorbidities have caused CKD, or CKD caused comorbidities, is debatable.

CVD, DM, and HT were more frequent in CKD patients compared to the control population. A study using medical history review of comorbidities in CKD patients estimated the ratio of DM and HT to be 32.4 and 66.8% respectively [23]. Another used the same ATC codes as our study to enhance ICD diagnosis of DM and HT resulting in 17% for both DM and HT and 31% with CVD [18]. In our cohort, DM was 17.2%, HT was 80.8% and CVD 29.0%. The increase of comorbidities along age groups is more pronounced in the control population. This may be the result of a high prevalence of comorbidity per se in the CKD group.

Diagnosis codes alone might lead to an underestimation of comorbidities as not all patients with HT or DM are registered as such in GP clinics. We argue that several drugs classified for HT, might be used for other purposes than lowering blood pressure and, therefore, might overestimate HT prevalence.

One UK study examining a cohort showed that low socioeconomic status is related to severity of CKD at presentation at nephrology outpatient clinics even when corrected for age and sex, supporting our findings [24]. One explanation could be that individuals with low socioeconomic status do not contact healthcare systems and, therefore, may present late in the disease. Whether the state of “not-economically-active” individuals is due to symptoms associated with CKD or other comorbidities, is unknown. Comparisons with other cohorts is complicated due to different classifications of educational levels [23].

The distribution of the initial CKD stages was comparable with previous studies with most cases present in the early stages [2, 18]. Age stratification across CKD stages showed that younger patients were more likely to become incident as CKD stage 5 patients or ESKD patients. This might reflect the more acute course of kidney disease in younger adults or may be due to the fact that creatinine testing in the younger population is sparse and probably only performed when patients feel ill Older patients may be more closely monitored and diagnosed earlier during their GFR decline.

Stage A1 was the most common albuminuria stage and A3 was more common in CKD stage 5. Treatment of albuminuria is important and screening for it is pivotal [6]. Assessment of albuminuria may be insufficient in our cohort since only albumin/creatinine ratios are presented and not urinary dipstick or 24-hour urine collection sample results. Awareness should be raised to screen for albuminuria with relevant tools and commence appropriate treatment should if indicated [18, 25].

A relatively stable prevalence since 2004 was described in USA in accordance with our findings [26]. The adjusted incidence rate of ESKD in USA has declined slightly since 2006 [26]. One study found an estimated 19.6% increase in CKD globally, when using a complex Bayesian model integrating multiple sources from 2005 to 2015 [27]. The increase of CKD was associated with aging of the global population. In our cohort, CKD incidence was above 50% in age groups 80+. An increasing number of patients in this age group would, likewise, cause an increase in CKD in our cohort. Furthermore, the question remains, how Covid-19 affects global CKD prevalence and incidence.

The varying results between studies may be explained by population representativeness, different biomarker essays [28], time window for included creatinine assessment [15], assessment of albuminuria [9], exclusion of creatinine measured during admission [18], and/or the use of different GFR equations [2]. Scandinavian studies using eGFR as a marker for CKD suggest a crude prevalence of 6.1% in the Stockholm area in Sweden and 4.1% in a cohort covering the island of Funen in Denmark [15, 18].

To ensure that kidney impairment was chronic, all eGFR measurements within the minimum period of 3 months had to be < 60 ml/min/1.73m^2^. The CKD-EPI equation used in our cohort is recommended by the KDIGO [6].

Coverage in our cohort was high at 78% [12]. Therefore, we maintain that our prevalence and incidence rates are reliable estimates of the true figures. Regarding the younger population and healthier individuals our data is less representative, as our study is based on general creatinine assessment which is not a part of a systematic screening program.

### Strengths

Our data not only presents CKD stage 3–5 prevalence and incidence data, but also presents data on demographics, comorbidity, albuminuria, and socioeconomic data for the entire KidDiCo. The study strictly follows KDIGO guideline criteria for CKD definition. High coverage underlines the representativeness of the study population.

### Limitations

Despite high coverage, the study design leads to selection bias since we only included patients with creatinine measurements. This patient group is older and, therefore, probably sicker than the general control population [12]. Therefore, we might underestimate prevalence and incidence in younger age groups. Furthermore, the data assumes that the control population not covered by the KidDiCo do not have CKD stage 3–5.We did not include patients with CKD stage 1–2. This was due to the sparse albuminuria screening which would have resulted in an underestimation of patients with CKD stage 1–2 and consequently, incorrect data. It is well known that GFR and thereby, CKD stages are fluctuant. Since we estimate the CKD stage according to the first measured GFR where the patients fulfil our inclusion criteria, we might have both under and/or overestimated the CKD stage in patients with acute kidney injury. As this goes both ways, we do not consider this a systematic error.

### Clinical perspective

Establishment of the prevalence and incidence of CKD stage 3–5 can help to optimize prevention strategies and public health measures. As CKD patients are at high risk for CVD, further studies regarding epidemiology, prevention, treatment strategies, interpretation of biomarkers etc. are needed.

## Conclusion

CKD stage 3–5 is a common disease, especially in the elderly. CKD stage 3–5 patients are predominantly women. KidDiCo data suggests an association between lower socioeconomic status and prevalence of CKD. Further research should examine socioeconomic status as a risk factor for CKD.

## Supplementary Information

**Additional file 1: Supplement Figure 1.** Overview of the overlap from the different data from KidDiCo and data from Statistics Denmark.

**Additional file 2: Supplement Table 1.** Sex stratification divided into CKD and control population.

## Data Availability

The datasets generated and/or analysed during the current study are not publicly available due Danish Data security laws, but are available from the corresponding author on reasonable request if approved by the Danish authorities.

## Abbreviations

CKD: Chronic kidney disease
ESKD: End stage kidney disease
KidDiCo: Kidney Disease Cohort
KDIGO: Kidney Disease: Improving Global Outcomes
GFR: Estimate glomerular filtration rate
CKD-EPI: Chronic Kidney Disease Epidemiology Collaboration
DNPR: Danish National Patient Register
ICKDD: Individual CKD date
FACM: First available creatinine measurement
DCRS: Danish Civil Registration System
DNPrR: The Danish National Prescription Registry
ATC: Anatomic Therapeutic Chemical-code
CS: Charlson score
DM: Diabetes mellitus
HT: Hypertension
CVD: Cardiovascular disease
NA: Not-active
SCREAM: Stockholm Creatinine Measurements project
