# Supplementary material for: Prevalence and incidence of chronic kidney disease stage 3–5 – results from KidDiCo
Source: BMC Nephrol. 2023 Jan 19;24:17. doi: 10.1186/s12882-023-03056-x (PMC9849831; doi:10.1186/s12882-023-03056-x)
Supplement: Supplementary file 1 — Additional file 1: Supplement Figure 1. Overview of the overlap from the different data from KidDiCo and data from Statistics Denmark. [file 12882_2023_3056_MOESM1_ESM.docx]

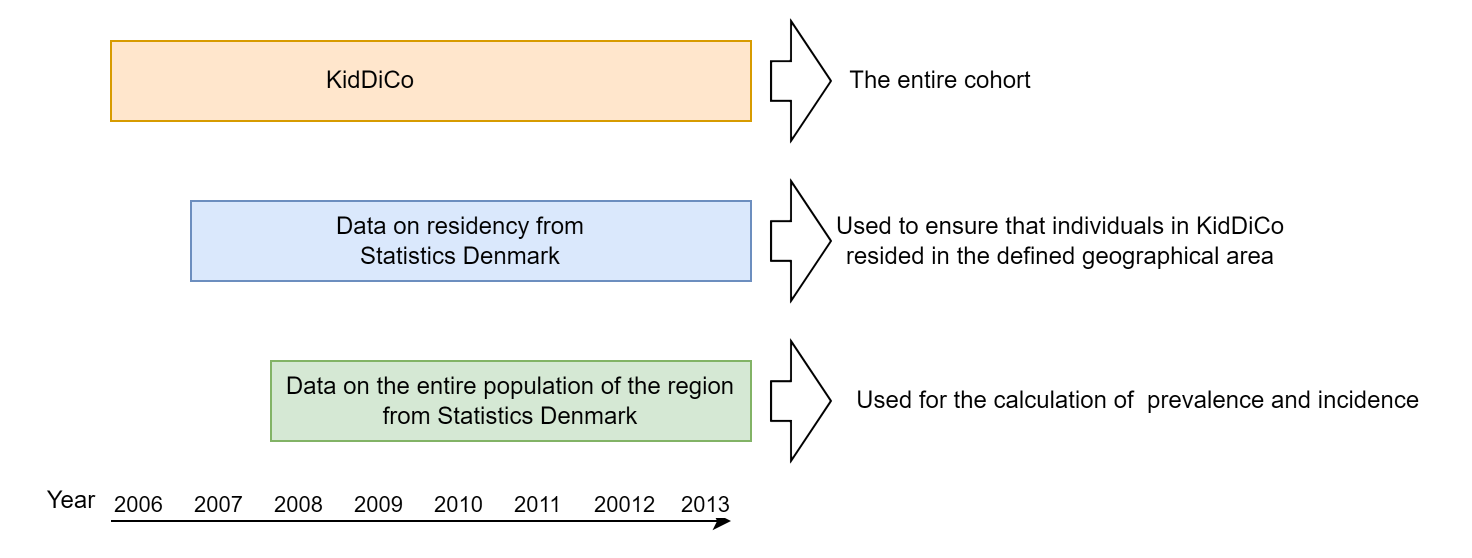


**Supplement Figure 1 overview of the overlap from the different data from KidDiCo and data from Statistics Denmark**
