# Supplementary material for: Prevalence and incidence of chronic kidney disease stage 3–5 – results from KidDiCo
Source: BMC Nephrol. 2023 Jan 19;24:17. doi: 10.1186/s12882-023-03056-x (PMC9849831; doi:10.1186/s12882-023-03056-x)
Supplement: Supplementary file 2 — Additional file 2: Supplement Table 1. Sex stratification divided into CKD and control population. [file 12882_2023_3056_MOESM2_ESM.docx]

**Supplement Table 1 Sex stratification divided into CKD and control population**

|  |  | CKD male | CKD female | Non-CKD male | Non-CKD female | p-value |
| --- | --- | --- | --- | --- | --- | --- |
|  |  | **N=26,305** | **N=40,181** | **N=283,452** | **N=319,991** |  |
| agegroup  stratification | 18-39 | 231 ( 0.9%) | 228 ( 0.6%) | 81,711 (28.8%) | 110,372 (34.5%) | <0.001 |
|  | 40-69 | 7,306 (27.8%) | 10,387 (25.9%) | 169,909 (59.9%) | 170,969 (53.4%) |  |
|  | 70+ | 18,768 (71.3%) | 29,566 (73.6%) | 31,832 (11.2%) | 38,650 (12.1%) |  |
| Diabetes | no | 20,831 (79.2%) | 34,249 (85.2%) | 269,506 (95.1%) | 310,050 (96.9%) | <0.001 |
|  | yes | 5,474 (20.8%) | 5,932 (14.8%) | 13,946 ( 4.9%) | 9,941 ( 3.1%) |  |
| Hypertension | no | 4,734 (18.0%) | 8,007 (19.9%) | 216,988 (76.6%) | 244,522 (76.4%) | <0.001 |
|  | yes | 21,571 (82.0%) | 32,174 (80.1%) | 66,464 (23.4%) | 75,469 (23.6%) |  |
| Cardiovascular diseases | no | 16,425 (62.4%) | 30,752 (76.5%) | 264,705 (93.4%) | 307,938 (96.2%) | <0.001 |
|  | yes | 9,880 (37.6%) | 9,429 (23.5%) | 18,747 ( 6.6%) | 12,053 ( 3.8%) |  |
| Charlson | 0 | 16,667 (63.4%) | 28,844 (71.8%) | 262,820 (92.7%) | 294,238 (92.0%) | <0.001 |
|  | 1 | 2,385 ( 9.1%) | 3,538 ( 8.8%) | 8,160 ( 2.9%) | 10,599 ( 3.3%) |  |
|  | 2 | 5,397 (20.5%) | 5,961 (14.8%) | 10,380 ( 3.7%) | 12,968 ( 4.1%) |  |
|  | 3 | 956 ( 3.6%) | 916 ( 2.3%) | 791 ( 0.3%) | 842 ( 0.3%) |  |
|  | 4+ | 900 ( 3.4%) | 922 ( 2.3%) | 1,301 ( 0.5%) | 1,344 ( 0.4%) |  |
| Education level | short | 20,762 (78.9%) | 29,896 (74.4%) | 222,992 (78.7%) | 213,750 (66.8%) | <0.001 |
|  | middle | 2,496 ( 9.5%) | 4,221 (10.5%) | 44,068 (15.5%) | 88,841 (27.8%) |  |
|  | long | 301 ( 1.1%) | 96 ( 0.2%) | 6,604 ( 2.3%) | 5,792 ( 1.8%) |  |
|  | missing | 2,746 (10.4%) | 5,968 (14.9%) | 9,788 ( 3.5%) | 11,608 ( 3.6%) |  |
| Occupational status | active | 2,604 ( 9.9%) | 2,208 ( 5.5%) | 163,117 (57.5%) | 159,429 (49.8%) | <0.001 |
|  | temporary not active | 144 ( 0.5%) | 147 ( 0.4%) | 6,951 ( 2.5%) | 9,720 ( 3.0%) |  |
|  | not active | 23,429 (89.1%) | 37,574 (93.5%) | 104,947 (37.0%) | 139,197 (43.5%) |  |
|  | missing/others | 128 ( 0.5%) | 252 ( 0.6%) | 8,437 ( 3.0%) | 11,645 ( 3.6%) |  |
